# Differences in Average Power Output Values from Computational Models of Repeated Vertical Jump Tests: A Single-Group Quasi Experimental Approach

**DOI:** 10.3390/jfmk10040397

**Published:** 2025-10-13

**Authors:** Vlad Adrian Geantă, Pierre Joseph de Hillerin, Alexandra Reta Iacobini, Carmen Magdalena Camenidis, Anca Ionescu

**Affiliations:** 1 Doctoral School of Sport Science and Physical Education, Pitesti University Center, National University of Science and Technology Politehnica Bucharest, 110040 Pitesti, Romania; hillerin@live.com (P.J.d.H.); alexandra.iacobini@spiruharet.ro (A.R.I.); mcamenidis@yahoo.com (C.M.C.); anca.ionescu@unitbv.ro (A.I.); 2 Department of Physical Education and Sport, Faculty of Physical Education and Sport, Aurel Vlaicu University of Arad, 310330 Arad, Romania; 3 Neuromotrica-Information for Sport and Human Performance Ltd., 021323 Bucharest, Romania; 4 Department of Physical Education and Sport, Faculty of Physical Education and Sport, Spiru Haret University, 030045 Bucharest, Romania; 5 Department of Motric Performance, Transylvania University of Brasov, 600115 Brașov, Romania

**Keywords:** vertical jump, lower limb power, athlete profiling, sport performance assessment

## Abstract

**Background:** Repeated vertical jump tests are widely used to assess neuromuscular function and lower limb performance. However, inconsistent formulas for average power output produce large discrepancies, limiting comparability across studies and limiting practical applications. This study aimed to compare three different models for the calculation of average power output, Bosco, Miron Georgescu (MG), and Modified Miron Georgescu-15s (MGM-15), applied to identical jump test data, in order to evaluate their computational behavior and practical relevance in athlete performance profiling. **Methods:** A single-group quasi-experimental study was conducted with 25 physically active male university students (mean age: 21.4 ± 2.7 years), who performed a 15 s repeated vertical jump test on the OptoJump Next system. Raw parameters including flight time, contact time, and jump height were recorded and exported. Average power output (W/kg) was subsequently calculated using three distinct computational models, each applied to the same dataset of flight and contact times. A repeated-measures ANOVA was used to compare outputs across models, with Bonferroni-adjusted pairwise comparisons for post hoc analysis (α = 0.05). **Results:** Significant differences were observed (*p* < 0.001). The Bosco model produced the highest values of average power (40.13 ± 8.56 W/kg), followed by MG (21.07 ± 5.92 W/kg), while MGM-15 yielded the lowest and most consistent outputs (4.08 ± 0.61 W/kg). Effect sizes were very large (η^2^_p_ = 0.952), confirming that calculation models strongly influenced the outcomes. **Conclusions:** The findings demonstrate that average power output differed markedly across formulas, despite identical performance data. Bosco and MG models tended to overestimate values due to simplified assumptions, whereas the MGM-15 method produced lower and more consistent outputs that may better capture repeated jump demands. The standardization of computational models is fundamental to ensure comparability and to improve athlete performance profiling in research and practice.

## 1. Introduction

Vertical jumps have long been regarded as one of the most practical field tests for assessing neuromuscular function and lower limb performance [1,2,3]. Their popularity comes from the simplicity of execution, non-invasive nature, and sensitivity to training-induced adaptations across many sports [4,5,6]. Distinct performance patterns are also observed in sports characterized by repeated, similar motor demands [7,8]. Recent evidence further supports their relevance, showing that jump-derived metrics are associated with independent measures of athletic performance [9].

The precision of jump testing has increased with modern technologies such as force plates and optical or inertial systems [10,11,12,13,14]. Among the available protocols, repeated jump tests are particularly informative, as they integrate both flight and contact times, providing a detailed evaluation of fatigue resistance, intermuscular coordination, and average power output [15,16,17,18,19,20]. Research shows these tests elicit changes in energetic and neuromuscular control parameters at the articular level, as well as in movement patterns, including the anticipatory dynamics of the muscular and tendinous structures involved [21,22]. Moreover, they can effectively track fatigue resistance and training adaptation across sport-specific contexts such as judo [23], volleyball [24], and football [25].

Historically, repeated jumps have often been interpreted as proxies for anaerobic capacity, echoing laboratory-based protocols such as the Wingate test [20,26]. Yet, this interpretation is debated, since the elastic energy used and movement efficiency can amplify apparent power output, suggesting that these tests reflect a broader neuromuscular strategy rather than pure energy system contribution [27].

Several computational models have been proposed to estimate average power output in repeated vertical jump assessments, each emerging from different theoretical premises. The first structured version, developed by the Romanian doctor Miron Georgescu (MG) in the 1950s, emphasized endurance-like jumping ability in a fixed time interval or fixed number of jumps, and is still known today in Latin America as the “Georgescu Test” [28]. The original protocol prescribed 35 continuous “ball like” jumps, from which aberrant ones were visually discarded, leaving the first 30 valid repetitions to be analyzed. To our knowledge, the MG protocol was also applied in Romania for the evaluation of various national elite sports teams before competitions [28]. Decades later, Bosco introduced a formula linking jump count and flight time, which spread internationally because of its operational simplicity and compatibility with early measurement systems [19]. Both protocols, developed by Georgescu and Bosco, required the athlete to jump as high as possible while minimizing ground contact time, essentially “ball-like jumps”, hence the term repeated vertical jumps [28,29].

Critiques, however, pointed out that these early approaches treat jump mechanics as a purely energetic processes and neglect crucial variables such as neuromotor control, anticipatory coordination, and elastic energy reutilization [29,30,31]. To address these issues, the Modified-Miron Georgescu-15 s protocol (MGM-15) was introduced as a more integrative method, factoring in both mechanical outputs and psycho–neuro–motor elements such as coordination and fatigue regulation [29,31]. The psycho–neuro–motor concept, described by Marin et al. [32], emphasizes a holistic integration of psychological, neural and motor mechanism within human performance training.

Recent evidence has shown [30,33] that the Bosco and MG models may produce substantially amplified values when elastic contributions dominate the movement, even in non-biological simulations (for example a repeated ball bounces). In contrast, MGM-15 provides more conservative estimates that appear better aligned with physiological plausibility [30]. Despite the widespread use of these formulas, the central challenge remains identifying which method best reflects true mechanical power output. Bosco’s formula for average power, although widely adopted, tends to systematically overestimate performance, a limitation that may compromise the accuracy of athlete monitoring and result interpretation [33]. More integrative models, such as MGM-15, could offer estimates that are biomechanically, physiologically, and from the psycho–neuro–motor perspective more consistent, accounting for mechanical outputs, anticipatory coordination, elastic energy use, and movement variability [19,20].

Although a preliminary investigation comparing these three methods under identical conditions [33] had a small sample (N = 5), it revealed significant discrepancies between models, and was the first to directly compare all three calculation methods. To strengthen the evidence, the present study extends the investigation to a larger cohort of subjects.

This study aims to determine whether these methodological differences persist in a larger sample. We hypothesize that significant differences will emerge between models, with MGM-15 offering the most functionally relevant estimates of lower limb power. These findings have practical implications for accurate performance monitoring and informed training decisions in sport performance.

## 2. Materials and Methods

### 2.1. Participants

Twenty-five physically active male university students (age, M = 21.4 ± 2.7 years; height, M = 178.6 ± 4.72 cm; body mass, M = 73 ± 8.12 kg), all enrolled at the Faculty of Physical Education and Sport, were recruited on a voluntary basis from various sport disciplines. To reduce potential selection bias, participants who met the inclusion and exclusion criteria were randomly chosen from the eligible pool until the target sample size was reached. Inclusion criteria required participants to be actively engaged in competitive sports training at least three times per week for the past six months, and to be familiar with performing vertical jumps. Exclusion criteria included any musculoskeletal injury or lower-limb surgery, as well as any cardiovascular, neurological, or balance-related disorders that could interfere with performance or safety. Only male athletes were included because the proportion of female athletes available in the faculty at the time of recruitment was very small, which would not have allowed for balanced representation or reliable statistical comparison. Elite athletes were also excluded in order to avoid heterogeneity due to highly specialized training backgrounds, which could have required stratified sampling and larger cohorts. Most participants reported a background in football training, while others were recreationally engaged in fitness or related activities, leading to a relatively homogeneous group in terms of performance level. These inclusion and exclusion criteria, as well as the preparticipation health screening process, were based on ACSM guidelines to ensure participant safety and methodological rigor [34], and are consistent with previous studies using vertical jump testing [35,36].

Participants were asked to refrain from intense physical activity for at least 24 h before testing and to maintain normal hydration and sleep routines. All participants provided written informed consent. The study protocol was approved by the institutional ethics committee (Registration number: 326/02.06.2025) and conducted in accordance with the Declaration of Helsinki.

### 2.2. Research Design

This study employed a single-group quasi-experimental design to investigate the variability of lower limb average power output (W/kg) calculated using three established models: Bosco [19], MG [28], and the MGM-15 proposed by Hillerin [29,31]. The goal was to compare the results yielded by each model during a standardized repeated vertical jump test performed over 15 s.

### 2.3. Procedure

Before starting the assessment, all participants were informed about the study procedures and objectives, and standardized demonstrations of the repeated jumps task were provided to ensure that they clearly understood the execution requirements. After this briefing, participants performed a standardized warm-up consisting of 5 min of light jogging, followed by dynamic stretching and three sets of submaximal countermovement jumps to prepare the neuromuscular system for maximal efforts. To ensure familiarization, all participants also performed submaximal practice trials of the repeated jump task during the standardized warm-up, following identical instructions as were set for the main test. This procedure minimized learning effects and ensured comparable technical execution. The warm-up protocol was designed to minimize injury risk and ensure consistent test conditions. All tests were conducted in a temperature-controlled indoor environment (20 °C, moderate ventilation) in accordance with ACSM facility standards [37]. This setting was selected to ensure safety, comfort, and standardized test conditions by minimizing environmental influence on neuromuscular performance.

**Figure 1:**
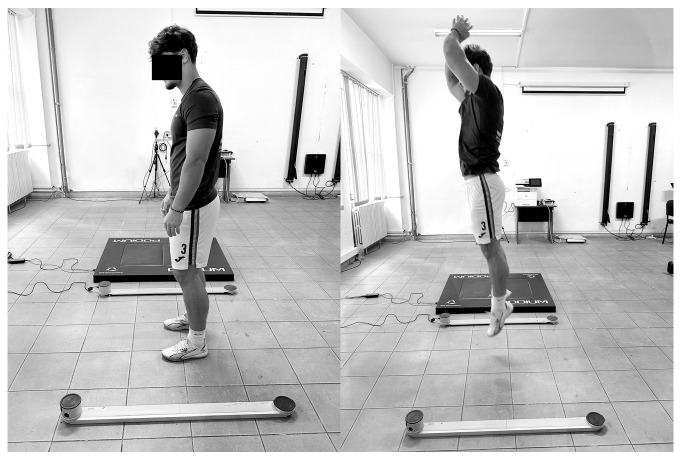
The 15 s repeated vertical jump test with arm swing.

To assess lower-limb average power output, participants performed maximal consecutive vertical jumps for 15 s (15 s jump test), using both legs and assisted by arm swing. This mode of jumping like a ball focuses on elastic energy return and neuromuscular efficiency. The test was administered using the OptoJump Next System (Microgate, Bolzano, Italy), a validated optical measurement device [38], which recorded jump metrics in real time and provided auditory signals marking the start and end of the trial (see Figure 1).

Participants were instructed to jump as high and as elastically as possible throughout the duration, minimizing ground contact time and maximizing flight time in each repetition.

OptoJump recorded raw data on flight time (T_f_), contact time (T_c_), and jump height (h), which were exported in XML format for analysis. Based on these parameters, three models were applied to calculate the average power output (PU) for each participant.

*MG methodology*

(1) $$\mathrm{PU}=1.5\;\times \;\frac{g^{2}\;\times \;{T_{f}}^{2}}{8\;\times T_{c}}$$

where, PU = average power output (W/kg), g = gravitational acceleration (typically 9.81 m/s^2^), T_f_ = flight time (s), and T_c_ = contact time (s). This method emphasizes the explosive efficiency by relating flight time squared to ground contact duration.

*Bosco methodology*

(2) PU=2×g2 ×
T
f ×154n × 15−Tf

where, n is the number of jumps in 15 s, PU = average power output (W/kg), g = gravitational acceleration (typically 9.81 m/s^2^), T_f_ = flight time (s), and T_c_ = contact time (s). This model uses cumulative flight time and jump count to estimate mechanical power.

*MGM-15 methodology *

(3) $$\mathrm{PU}=\frac{g^{2}\times \;{T_{f}}^{2}}{8\times \left(T_{c}+T_{f}\right)}$$

where, PU = average power output (W/kg), g = gravitational acceleration (typically 9.81 m/s^2^), T_f_ = flight time (s), and T_c_ = contact time (s). This updated formula balances both flight and contact time, offering a more conservative and time-specific representation of lower limb power.

In all three models, g = 9.81 m/s^2^ (acceleration due to gravity). By applying these formulas to the same jump performance data, this design allowed for direct comparisons and the identification of systematic discrepancies among the models, with implications for performance assessment accuracy.

### 2.4. Worked Example

For clarity and replicability, we provide here a worked example of how Equation (3) was applied to the raw OptoJump data. The system exports for each jump the flight time (T_f_) and contact time (T_c_), from which jump height (m) is also derived. For instance, in one jump, the parameters were T_c_ = 0.223 s T_f_ = 0.565 s, corresponding to a jump height of 39.1 cm (0.391 m). Substituting into Equation (3),

$$\mathrm{PU}=\frac{9.81^{2}\times {\left(0.565\right)}^{2}}{8\times \left(0.223+0.565\right)}=\frac{96.24\times 0.319}{8\;\times 0.788}=\frac{30.72}{6.30}=4.88\;W/\mathrm{kg}$$

Applying the same computation to the following jump (T_c_ = 0.202 s, T_f_ = 0.538 s, jump height 35.5 cm = 0.355 m) yields a value of 4.37 W/kg. This procedure was repeated for every jump recorded in the 15 s test series, and the arithmetic mean of all average power values represents the final MGM-15 output for each participant. The calculations can be replicated directly using the raw XML export from OptoJump, which contains all required kinematic parameters. The worked example is provided for illustration purposes; the same procedure was applied to all jumps in each 15 s series to calculate the final values reported in this study.

### 2.5. Statistical Analysis

Descriptive statistics (mean and standard deviation) were calculated for each of the three average power output calculation models (Bosco, Miron Georgescu, and MGM-15) to provide an overview of data distribution and variability. The normality of each variable was assessed using the Shapiro–Wilk test, which is appropriate for small to moderate sample sizes. Since all variables met the assumption of normal distribution (*p* > 0.05), parametric tests were applied in subsequent analyses.

Therefore, to compare average power output values across the three methods, a one-way repeated-measures analysis of variance (ANOVA) was applied, suitable for analyzing differences within subjects across multiple conditions [39]. To evaluate whether the assumption of sphericity was met for the repeated-measures ANOVA, Mauchly’s test of sphericity was conducted. In cases where this assumption was violated, the Greenhouse–Geisser correction was used to adjust the degrees of freedom and preserve the validity of the statistical inference. Following a significant main effect, post hoc pairwise comparisons were performed using Bonferroni-adjusted paired *t*-tests, which correct for the Type I error rate associated with multiple comparisons. Effect sizes were reported using partial eta squared (η^2^_p_), with interpretation thresholds based on Cohen’s conventional values [40]: small (≥0.01), medium (≥0.06), and large (≥0.14).

All statistical tests were two-tailed, and the significance level was set at *p* < 0.05. Data analysis was conducted using IBM SPSS Statistics for Windows, version 23.0 (IBM Corp., Armonk, NY, USA).

## 3. Results

Descriptive statistics, assumption testing, and results from the repeated measures ANOVA are presented below. Statistically significant differences between the three methods of average power calculation (Bosco, MG, MGM-15) were identified. Detailed results are provided in Table 1, Table 2, Table 3 and Table 4.

An examination of the descriptive statistics (Table 1) shows the highest mean power output was derived using the Bosco formula (M = 40.13 ± 8.56 W/kg), followed by the MG formula (M = 21.07 ± 5.92 W/kg). The lowest values were observed using the MGM-15 method (M = 4.08 ± 0.61 W/kg). On the other hand, the standard deviation was much lower for MGM-15 (SD = 0.61 W/kg) compared to both Bosco (SD = 8.56 W/kg) and MG (SD = 5.92 W/kg).

**Figure 2:**
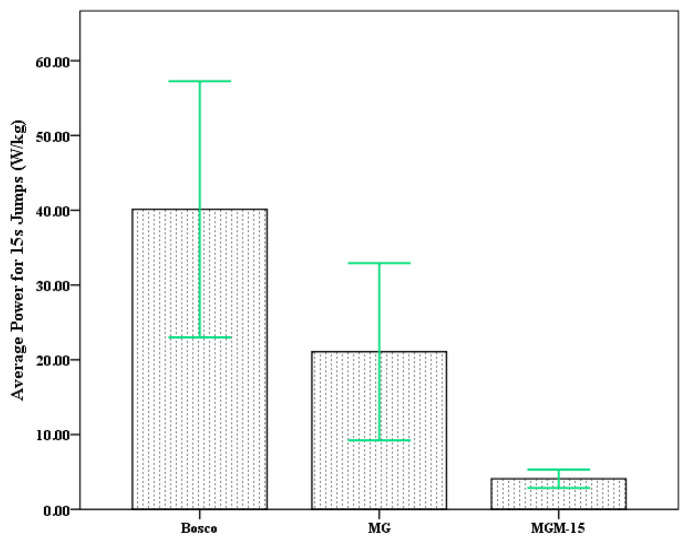
Differences in average power output between models.

This narrower dispersion suggests that MGM-15 produced more consistent outputs across participants, potentially indicating reduced sensitivity to inter-individual variation or to small fluctuations in flight and contact time. Given that all models were applied to the same jump data, this discrepancy in variability underscores fundamental differences in how each method weights biomechanical components such as flight time or contact duration (see Figure 2).

According to Table 2, Mauchly’s test indicates that the assumption of sphericity was violated (*p* = 0.001), confirming that the variances of the differences between conditions were not equal. This violation further highlights that the three computational models produce not only different central tendencies, but also diverging variance structures, despite being applied to a single within-subjects dataset. The Greenhouse–Geisser correction (ε = 0.531) was therefore applied in all subsequent analyses to reduce the risk of Type I error.

The corrected repeated measures ANOVA from Table 3 reveals a statistically significant main effect of the calculation method on average power output, F (1.06, 25.47) = 479.89, *p* < 0.001, with a very large effect size (η^2^_p_ = 0.952). This indicates that the differences between methods were systematic and not random. The magnitude of the effect reflects the strong influence of the computational model on the resulting values, even though the physical performance data remained constant. Thus, the formulas used above explain this outcome. For example, the Bosco model multiplies cumulative flight time and uses jump count, magnifying the results; MG uses a multiplier of 1.5 and disregards flight–contact balance, whereas MGM-15 integrates both flight time (T_f_) and contact time (T_c_), reducing overestimation.

Pairwise comparisons confirm that all differences between methods were statistically significant, even after correcting for multiple comparisons (*p* < 0.001 in all cases). The Bosco model produced values nearly ten times higher than MGM-15, while the MG formula occupied an intermediate position.

## 4. Discussion

This study aimed to determine whether three commonly used models for estimating average power output during repeated vertical jump tests (Bosco, MG, and MGM-15) yield significantly different values when applied to identical performance data. Our findings clearly confirm this hypothesis, revealing systematic and statistically significant discrepancies across all calculation models. Among them, the Bosco formula produced the highest power estimates, followed by the MG model, while MGM-15 consistently reported the lowest values.

Beyond the statistical confirmation, the magnitude and practical implications of these discrepancies are noteworthy, as they could lead to opposite conclusions about an athlete’s power profile depending on the formula applied. These differences arise not from performance variation, but from the mathematical structure of each formula, particularly in how they integrate (or omit) kinematic parameters such as contact time, flight time, and temporal distribution across repetitions. The observed discrepancies may also be amplified by the specific characteristics of the jumping protocol used in this study. The repeated jumps performed “like a ball,” with maximal height and minimal contact time, emphasize elastic energy return. Some formulas do not explicitly account for this component, potentially contributing to the variability and inflation observed in their average power outputs.

These differences were not only statistically robust (*p* < 0.001 for all pairwise comparisons), but were also practically significant, as demonstrated by a large effect size (partial η^2^_p_ = 0.952). Importantly, this robustness does not reflect novel evidence of superior physiological performance, but rather represents a new empirical confirmation that the Bosco and MG formulas systematically overestimate average power, in contrast to the more conservative MGM-15 model. Our study does not assume that these discrepancies represent differences in real functional capacity; rather, they stem from the structural characteristics and underlying assumptions of the respective formulas. Given the relatively small sample size, these findings should be interpreted with caution. The 36.04 W/kg gap between Bosco and MGM-15 underscores that these models are not interchangeable representations of the same construct, but rather embody fundamentally divergent assumptions about what is being calculated [33].

Notably, both Miron Georgescu and Carmelo Bosco adopted assumptions that mathematically amplified the calculated power output. Georgescu, in his 1953 article, proposed a dual-phase contact model—a passive “damping” phase consuming half of the necessary energy, followed by an active contraction phase producing the equivalent of m × g × h. This reasoning justified his use of a 1.5 multiplication factor in the formula. Bosco, by contrast, applied an even more inflationary factor of 2, and additionally divided solely by contact time, thereby implicitly treating the muscular system as a continuous-output engine while disregarding the elastic contribution [30]. However, this interpretation oversimplifies the real biomechanics of muscle contraction. The mechanical cycle of repeated jumping may be better conceptualized as a two-phase system, where both contraction and relaxation phases contribute to the output, rather than being reduced solely to the propulsion phase [33]. In fact, during the flight phase, the muscle continues to function in the short time of relaxation similarly to a two-stroke engine, so the flight time must also be considered as part of the complete movement cycle. Calculating muscular power based only on the brief propulsion phase neglects contributions from elastic and neuromuscular mechanisms, thus potentially overestimating actual performance [30,31].

The Bosco method of repeated vertical jumps, originally designed in 1983 [19] for rapid field application, relies heavily on cumulative flight time and jump count. Its simplicity has contributed to its widespread adoption in performance diagnostics. However, as demonstrated in our results and supported by prior literature [33,41], this approach may conflate mechanical displacement with muscular output. The elevated values generated by the Bosco formula may reflect not only genuine explosive power, but also the influence of passive elastic recoil mechanisms, which are not accounted for in its calculation logic [27,42,43]. This limitation becomes even more pronounced when analyzing jump protocols that emphasize minimal ground contact and high-frequency execution, such as the “ball-like” style of jumping behavior that naturally exploits the stretch-shortening cycle, as advocated in Bosco-type tests [29,30]. These outcomes resonate with recent findings [33,44,45], reinforcing concerns that Bosco-derived values may exceed physiological plausibility, particularly under protocols favoring elastic return.

Similarly, the MG formula, which links squared flight time to contact time, offers a closer approximation to explosive performance, but still fails to integrate factors such as neuromuscular coordination, fatigue progression, or energy utilization through the muscle–tendon unit [29,30,31,33,46]. Its intermediate results, while more conservative than Bosco, still appear to overestimate actual physiological output. Like Bosco, the MG model assumes that all elevation during the flight phase is the result of metabolic effort, ignoring the viscoelastic contributions of the body [33].

In contrast to these energetically amplified models, the MGM-15 method reduces the tendency to overestimate power output by integrating both flight and contact time, aligning with the shift toward evaluating jump tests as indicators of psycho–neuro–motor integrity rather than purely mechanical output [29,31]. Its structure accounts for both explosive capacity and efficiency in force transmission, making it particularly relevant when assessing neuromuscular readiness under fatigue conditions. Furthermore, the model’s compatibility with time-domain performance analysis complements prior work on neuromuscular anticipation and preparatory motor mechanisms [21,22].

From a practical standpoint, the underutilization of MGM-15 in international literature remains a gap that should be addressed. Despite its application in disciplines such as volleyball, football, judo, basketball, gymnastics, dance, handball, athletics [47,48,49,50,51,52,53,54,55,56,57,58], and other motor actions [59,60], its validation against gold-standard biomechanical tools is still limited. Comparative studies across various sports and athlete populations would enhance the methodological visibility and adoption of MGM-15.

An additional theoretical consideration is related to the limitations of traditional formulas when applied to elastic rebound. A previous control experiment [61] with a non-biological system (a basketball) demonstrated that the Bosco and MG formulas produce implausibly high energy outputs, despite the absence of metabolic or neuromuscular contributions [30]. This previous observation can be understood considering Pooper’s notion of a “critical experiment” [62], since it illustrates the falsification of the assumption that these models strictly quantify muscular power. Building on this earlier critique, the present findings reinforce the fact that elevated values in human jump tests may largely reflect elastic return [63,64] and computational assumptions [65] rather than true muscular power. Consequently, repeated jump protocols should not be interpreted solely as a measure of anaerobic capacity [66], but rather as integrative assessments of neuromuscular coordination and elastic efficiency [67], as well as fatigue regulation [26], constructs more accurately captured by the MGM-15 approach [30,31,33].

This finding is particularly relevant when considering that the human movement pattern in repeated jumps mimics elastic rebound. Participants are often instructed to minimize ground contact and maximize jump frequency behavior that naturally exploits the stretch-shortening cycle [63,64,65]. In such scenarios, mechanical output does not directly equate to energy expenditure, calling into question the traditional classification of such protocols as valid assessments of anaerobic power capacity [20,26,66,68,69].

Consequently, while our findings are statistically robust, they should not be interpreted as novel empirical confirmation of athletic superiority or deficiency, but rather as methodological evidence that computational assumptions drive the discrepancies. This aligns with prior critiques [33], which have demonstrated that overestimated outputs may result from the formula’s structure rather than genuine neuromuscular performance. Evidently, further research is needed. However, this study represents further incremental steps along this pathway, as part of our ongoing project on methodological standardization in jump-based performance diagnostics.

Thus, the common classification of these tests as “anaerobic power assessments” warrants reconsideration. Instead of serving as direct proxies for metabolic energy systems, the resulting values may reflect a complex interplay between motor control, elastic rebound, and rhythmic regulation, dimensions that align more closely with the conceptual framework underpinning the MGM-15 model [31]. The test evaluates not just how high or fast an athlete can jump, but how efficiently they can regulate repeated efforts through anticipatory coordination and fatigue-resistant patterns.

While the OptoJump Next system offers practical advantages in field-based performance diagnostics, the absence of simultaneous validation against a force platform in our study represents a methodological limitation. Although prior studies have demonstrated its validity and reliability in standard jump assessments, recent findings [16,65] highlight potential systematic biases, especially in high-frequency jump protocols where elastic energy contributions are prominent. Moreover, intra-session reliability was not directly assessed in our protocol. Given that our test involved repeated jumps, future research should specifically evaluate the reproducibility of OptoJump measurements in such a dynamic context.

### 4.1. Practical Implications

The accurate interpretation of power output is critical in athletic performance diagnostics, as it informs both immediate training decisions and long-term performance projections. When overestimated, such as through the application of the Bosco or MG models, power values may give coaches and athletes an overly optimistic representation of the individual’s current physical state and future potential. This potential issue can result in incorrect training loads, flawed recovery planning, and unrealistic expectations regarding progression. A limited ability to differentiate between genuine muscular effort and mechanically influenced output highlights notable methodological limitation in the widespread adoption of certain field tests. This trend may reflect an overemphasis on quantitative outcomes, sometimes at the expense of a deeper consideration of the underlying biomechanical and physiological mechanisms involved. The widespread use of such protocols, despite their conceptual shortcomings, underscores the need for a critical evaluation of what is being measured. The MGM-15 model’s conceptual alignment with motor control theories also makes it a viable candidate for integration into modern athlete monitoring systems, particularly in sports that prioritize movement quality and resilience under fatigue, such as gymnastics, martial arts, or team sports requiring repeated submaximal efforts [7,8,22]. Although currently its use is widespread mainly at the national level, its limited international adoption should not be interpreted as a methodological weakness, but rather as an underexplored area in the literature. Moreover, the MGM-15’s sensitivity to subtle neuromotor variations suggests its potential role in the early detection of overtraining or asymmetries, supporting individualized monitoring strategies [70]. Therefore, the careful differentiation between genuine physiological outputs and mechanically influenced values is important for ethical, data-informed, and individualized practice in sport science.

### 4.2. Study Limitations

While this study provides valuable insights, it has several limitations. First, the design was single-group quasi-experimental. All participants underwent the same procedure without the inclusion of a control group, which constitutes a fundamental limitation and prevents causal inferences from the observed outcomes. Second, the present study included only young, physically active male students enrolled at the Faculty of Physical Education and Sports. This restricts the generalizability of the findings to female athletes, older populations, or elite athletes with highly specialized training backgrounds. Although participants were recruited from different sport disciplines, the limited sample size prevented stratifications or comparative analyses across sports. Furthermore, sport-specific influences on repeated-jump behavior were not analyzed, as the primary aim of this study was to compare computational models rather than differences between disciplines. This aspect will be addressed in future phases of our project involving larger, multi-sport cohorts. This focus was deliberate, as recruiting physically active students of similar ages and backgrounds provided a relatively homogeneous sample, reducing inter-individual variability and allowing clearer methodological comparisons. However, this also limits external validity, particularly with respect to elite or female athletes. Third, although the repeated jump test is widely employed in sports diagnostics, the exclusive use of the OptoJump Next system without direct comparison to a force platform (gold standard) limits the ability to establish definitive physiological relevance for the proposed models. Previous research has shown that OptoJump provides valid and reliable results [10,71,72,73,74]. However, other studies have reported systematic bias when comparing OptoJump to force plates [16,65,75] particularly in tasks involving high-frequency jumping or elastic contributions. Therefore, while the device offers practical advantages in field testing, the lack of gold-standard validation in our specific protocol represents a limitation. Fourth, the intra-session reliability of the device was not assessed in the present study, which may represent a source of measurement error. Although prior studies have shown high intra-session and inter-session reliability for OptoJump [71,72,73,74], these findings may not fully generalize to repeated vertical jumps, as used in our protocol. Future studies should include test–retest analyses to better establish reproducibility. Furthermore, inter-rater reliability was not formally assessed; however, to reduce evaluator-related variability, all assessments were consistently supervised by the same research team under standardized conditions. Future phases of our project are specifically designed to address both test–retest and inter-rater reliability in repeated jump conditions. Fifth, although all participants received standardized instructions, demonstrations, and familiarization trials before testing, subtle individual differences in execution may still have influence jump mechanics and power estimation. In addition, fatigue control relied only on 24 h abstinence from intense activity, without objective markers such as ratings of perceived exertion (RPE) or heart rate (HR), which could introduce variability. Sixth, the absence of a longitudinal design precluded the evaluation of the sensitivity of each computational model to training-induced changes or long-term adaptations. Seventh, several important aspects were not controlled in the present study. Participants’ motivation and potential learning effects across repetitions were not objectively assessed, which may have influenced performance. Intra-jump variability, such as the dispersion of flight and contact times across the 15 s series, was also not analyzed, although this could provide valuable insights into neuromuscular control. Finally, although the use of arm swing was standardized in the instructions, subtle technical differences between participants may still have affected the results.

Taken together, these limitations underline the need for the cautious interpretation of the present findings, and highlight the importance of methodological standardization in future research.

## 5. Conclusions

This study shows that the estimation of average power output in repeated vertical jump tests depends strongly on the calculation model. The Bosco and MG formulas produced substantially higher values, reflecting assumptions that enhance mechanical output, while the MGM-15 method generated more conservative and biomechanically plausible results by integrating both flight and contact times. These discrepancies suggest that repeated jump tests reflect combined neuromuscular and elastic contributions, rather than true muscular energy expenditure. Importantly, by expanding on a preliminary investigation [33], our larger-sample analysis enhances the generalizability of these findings. Therefore, coaches and performance centers should be cautious, standardize computation methods, and consider MGM-15 as a more realistic framework for monitoring performance in applied sport science.

## Figures and Tables

**Table 1:** Descriptive statistics for average power output (N = 25).

Variable	M	SD
PU Bosco (W/kg)	40.13	8.56
PU MG (W/kg)	21.07	5.92
PU MGM-15 (W/kg)	4.08	0.61

Note. PU = average power; Bosco = Bosco formula; MG = Miron Georgescu formula; MGM-15 = Miron Georgescu Modified 15 s formula; W/kg = watts per kilogram.

**Table 2:** Mauchly’s test of sphericity and Greenhouse–Geisser correction.

Effect	W	χ^2^	df	*p*	ε (G-G)
Average Power	0.116	49.56	2	0.001	0.531

Note. W = Mauchly’s W statistic. ε (G-G) = Greenhouse–Geisser epsilon used for degrees of freedom correction due to violation of sphericity.

**Table 3:** Repeated-measures ANOVA for average power output.

Source	df	F	*p*	η^2^_p_
Calculation Model	1.06, 25.47	479.89	<0.001	0.952

Note. Greenhouse–Geisser-corrected degrees of freedom are reported due to the violation of sphericity (see Table 2). η^2^_p_ indicates effect size.

**Table 4:** Pairwise comparisons between conditions with Bonferroni adjustment.

**Comparisons**	**Mean Difference**	**SE**	***p***	**CI 95%**
Bosco vs. MG	19.05 *	0.61	0.001	[17.47, 20.63]
Bosco vs. MGM-15	36.04 *	1.59	0.001	[31.93, 40.15]
MG vs. MGM-15	16.99 *	1.06	0.001	[14.24, 19.73]

Pairwise comparisons were adjusted using the Bonferroni correction. SE = standard error; CI = confidence interval. * Significant at *p* < 0.05.

## Data Availability

Data are contained within the article.

## Abbreviations

The following abbreviations are used in this manuscript:

MG	Miron Georgescu
MGM-15	Modified Miron Georgescu 15 s
W/kg	Watts per kilogram
PU	Average power
T_f_	Flight time
T_c_	Contact time
g^2^	Gravitational acceleration
h	Jump height
